# Study of Hypersensitivity to *Malassezia furfur* in Patients with Atopic Dermatitis with Head and Neck Pattern: Is It Useful as a Biomarker and Therapeutic Indicator in These Patients?

**DOI:** 10.3390/life12020299

**Published:** 2022-02-16

**Authors:** Francisco José Navarro-Triviño, Ángela Ayén-Rodríguez

**Affiliations:** Department of Contact Eczema and Immunoallergic Diseases, Dermatology, Hospital Universitario San Cecilio, Avenida de la Investigación s/n, 18016 Granada, Spain; aayenrodriguez@gmail.com

**Keywords:** atopic dermatitis, dupilumab, *Malassezia furfur*, immunoglobulin E, itraconazole, head and neck pattern, ketoconazole, fluconazole

## Abstract

Atopic dermatitis (AD) is one of the most prevalent chronic inflammatory diseases. Head and neck (H&N) involvement, also known as the picture-frame pattern, can be a diagnostic and even therapeutic challenge. Sensitization to the fungus *Malassezia furfur* seems to be implicated in this clinical presentation. To investigate the role of *Malassezia furfur* in H&N dermatitis, we performed an observational single-centre study. Serum-specific IgE levels for *Malassezia furfur* were determined in a total of 25 patients with AD (15 receiving dupilumab treatment, 10 not receiving dupilumab), 14 patients with seborrheic dermatitis, and 19 healthy controls. Reactivity to *Malassezia* *furfur*, in terms of serum-specific IgE levels (>0.35 Ku.arb./L), was found in 80% of patients with AD. Risk factors to consider include high total IgE levels, sensitization to multiple pneumoallergens, and elevated LDH and CRP levels. Prescription of topical antifungals, oral antifungals, or a combination of both showed good response in 100% of cases in the H&N AD group treated with dupilumab. The most appropriate treatment seems to be the use of oral itraconazole and/or ketoconazole cream. The median treatment time was 3 weeks. Localized dermatitis in H&N significantly affects the patient’s life. We present a study of sensitization to *Malassezia furfur* in patients with H&N AD. It is important to know the differential diagnosis and to approach the study correctly. Sensitization to *Malassezia furfur* may be one of the main reasons, especially in patients being treated with dupilumab. The use of antifungals allows for adequate control, avoiding treatment changes and improving the patient’s quality of life.

## 1. Introduction

Atopic dermatitis (AD) is one of the most prevalent chronic inflammatory diseases [1]. Head and neck (H&N) involvement, also known as portrait dermatitis [2], can be a diagnostic and even therapeutic challenge in some patients [3]. Since the introduction of dupilumab in 2017 for the treatment of adult severe AD, several adult-specific articles on this pattern have been published. In addition, this location of eczema significantly compromises the quality of life of patients with AD [4]. Experience of H&N AD that does not improve with dupilumab in real-world clinical practice may be a reason to discontinue biologic therapy. Sensitization to the fungus *Malassezia furfur* seems to be implicated in this clinical presentation [5,6]. Based on this background, the aim of the present study was to investigate the role of specific IgE for *Malassezia furfur* in patients with AD, mainly as a cause of H&N pattern.

## 2. Material and Methods

We performed a single-centre observational pilot study at the Department of Contact Eczema and Immunoallergy of the Hospital Universitario San Cecilio de Granada, Spain. Patient recruitment took place from 1 February 2020 to 30 June 2020. Inclusion criteria were patients over 18 years of age diagnosed with AD who were under follow-up and treatment in our department. Exclusion criteria were refusal to participate in the study, refusal to take the blood test for the determination of specific IgE, and current antifungal treatment (both oral and topical) for another dermatological disease. Demographic, clinical, and serological data collected were patient age, sex, H&N involvement, total IgE serum levels, IgE levels for *Malassezia furfur*, lactate dehydrogenase (LDH) levels, and C-reactive protein (CRP) levels (Table 1).

### Statistical Analysis

Data were expressed as the mean and standard deviation (SD) for quantitative continuous variables or as proportions—expressed as percentages—for categorical variables. Associations between categorical variables were assessed using Fisher’s exact test. A Kruskal–Wallis test was performed for continuous variables across the different groups. Correlations between IgE levels for *Malassezia furfur* and other serological quantitative variables were assessed using Spearman’s rank correlation coefficient analysis. *p*-values < 0.05 were considered statistically significant. All statistical analyses were performed using Stata statistical software (V6.1; StataCorp, College Station, TX, USA).

## 3. Results

Table 1 shows the demographic, clinical, and serological characteristics of the patients included in the study. A total of 25 patients with AD (15 receiving dupilumab therapy), 14 patients with seborrheic dermatitis, and 19 healthy controls were enrolled in the study. The mean age of the atopic dermatitis group was 31.92 ± 10.26, 38.64 ± 17.08 for the SD group, and 36.58 ± 13.32 for the healthy group. The M/F sex ratio was 0.48 for the AD group, 0.79 for the SD group, and 0.63 for the healthy group. This difference was significant between patients with SD and healthy controls. The mean weight of the AD group was 73.24 ± 16.22, 82.86 ± 4.88 for the SD group, and 72.11 ± 9.78 for the healthy group. The three groups were comparable with each other in age, sex, and weight. H&N pattern involvement was observed in 68% of all AD patients, whereas 100% of DS patients showed H&N involvement. No patients in the healthy group showed H&N involvement. The difference was statistically significant among all the groups. Within the group of patients with atopic dermatitis, 93.3% of patients on dupilumab treatment showed hypersensitivity to *Malassezia furfur*, versus 60% of patients with atopic dermatitis without dupilumab treatment. However, this difference was not significant (*p* > 0.05). The prevalence of asthma and pneumoallergen positivity was significantly higher in patients with AD than in patients with DS and healthy patients, with no differences between the latter two groups. Among AD patients, 52% had associated asthma, 5% of patients in the healthy group had associated asthma, whereas none of the patients in the SD group had associated asthma. Pneumoallergen sensitization was detected in 88% of patients in the AD group, 15% in the SD group, and no patients in the healthy group showed sensitization to pneumoallergens. Total IgE levels in serum were 2210.96 ± 3260.30 for the AD group, 241.37 ± 481.33 for the SD group, and 36.3 ± 54.56 for the healthy group, showing significance differences between the three groups. Specific IgE determination against *Malassezia furfur* (considered positive with a value equal to or higher than 0.35 Ku.arb./L) was 80% in the AD group, whereas no patient in the SD or healthy group showed specific IgE positivity. With regard to blood inflammatory markers, LDH levels were 211.44 ± 53.62 for the AD group, 205.33 ± 35.20 for the SD group, and 189.83 ± 21.71 for the healthy group. Although no statistically significant differences were found between the groups, AD patients showed higher mean LDH levels than the other groups. Total CRP was 1.44 ± 0.82 for the AD group, 3.08 ± 2.40 for the SD group, and 1.96 ± 0.99 for the healthy group. No statistical differences were detected for CRP levels between the groups.

Table 2 shows these same characteristics within patients with atopic dermatitis after being divided into two groups according to whether or not they were under treatment with dupilumab. These two subgroups show significant differences in two parameters, weight and H&N involvement, affecting 100% of patients on dupilumab versus only 20% of those not on dupilumab.

**Figure 1:**
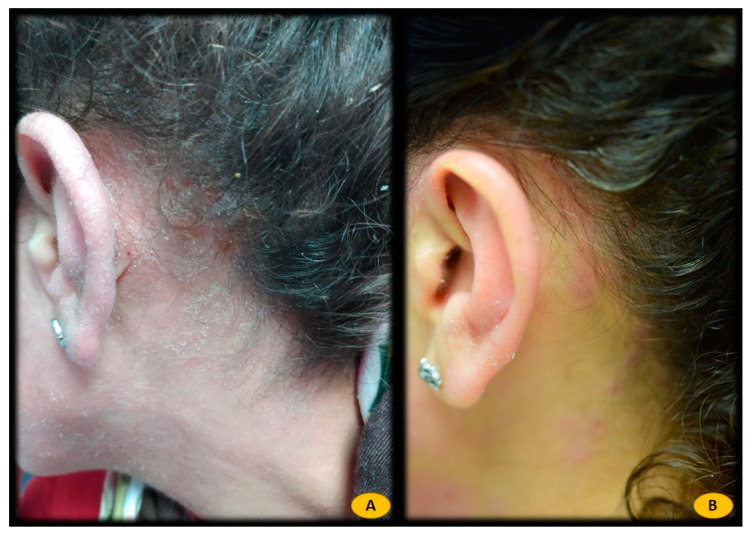
(**A**) Patient with atopic dermatitis H&N pattern treated with dupilumab. Positive IgE for *Malassezia furfur* (17.40 Ku.arb./L). (**B**) After 3 weeks of treatment with itraconazole 100 mg/12 h oral, almost complete response to treatment. *Malassezia furfur*-specific IgE levels of 1.2 Ku.arb./L.

**Figure 2:**
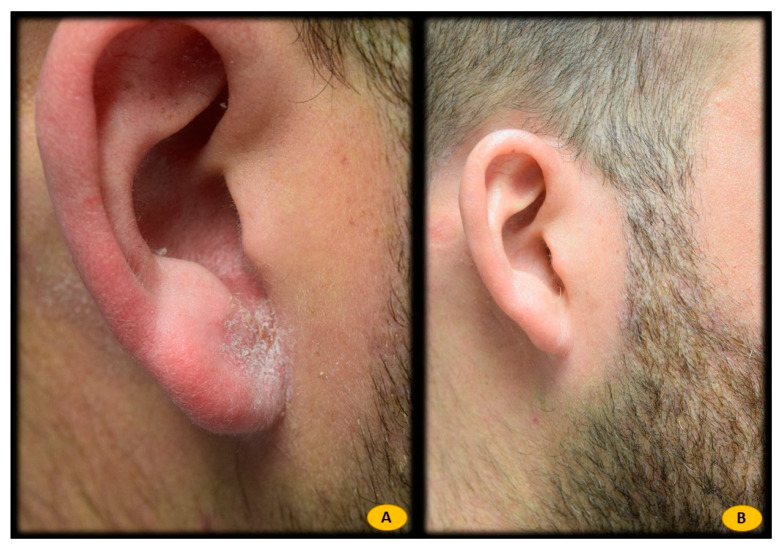
(**A**) Patient with atopic dermatitis H&N pattern treated with dupilumab. Positive IgE for *Malassezia furfur* (0.88 Ku.arb./L). (**B**) After 3 weeks of treatment with ketoconazole 2% cream every 12 h, complete response to treatment. *Malassezia furfur*-specific IgE levels < 0.1 Ku.arb./L.

**Figure 3:**
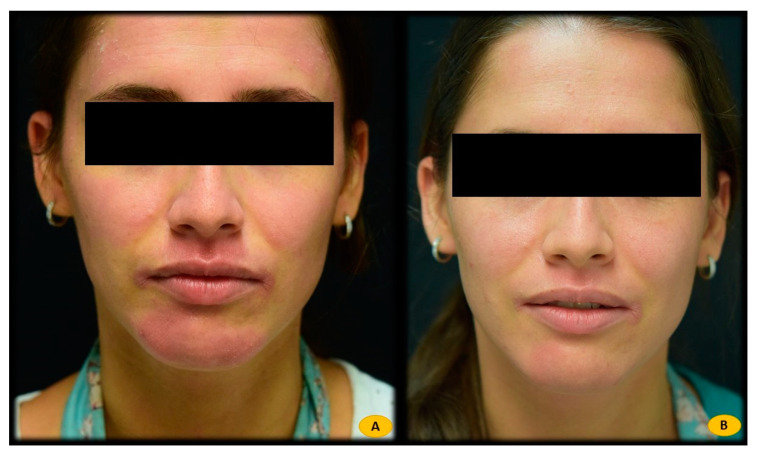
(**A**) Patient with atopic dermatitis H&N pattern treated with dupilumab. Positive IgE for *Malassezia furfur* (48.80 Ku.arb./L). (**B**) After 3 weeks of treatment with itraconazole 100 mg/12 h oral and ketoconazole 2% cream every 12 h, complete response to treatment. *Malassezia furfur*-specific IgE levels of 12.2 Ku.arb./L.

Statistical analysis showed a statistically significant correlation between *Malassezia furfur*-specific IgE levels and the following serological variables: elevated total IgE levels (*p* = 0.000), sensitization to different pneumoallergens (*p* < 0.001), and elevated CRP levels (*p* = 0.010). In addition, this positive association was also found with current treatment with dupilumab (*p* = 0.001). However, no significant relationship was found between *Malassezia furfur*-specific IgE levels and variables such as sex, severity of AD (measured by the Eczema Area and Severity Index (EASI), body surface area (BSA), and Investigator Global Assessment (IGA)), personal history of asthma or LDH levels. Patients with H&N pattern AD and positive *Malassezia furfur*-specific IgE (n = 16) were treated with 100 mg of itraconazole every 12 h for 3 weeks (n = 2) (Figure 1), ketoconazole 2% cream every 12 h for 3 weeks (n = 3) (Figure 2), or both therapies (n = 11) (Figure 3), depending on the extent and severity of the lesions.

## 4. Discussion

Blocking the Th2 pathway using biological therapies such as dupilumab is a successful therapeutic strategy in patients with AD [7]. This type of inflammatory pathway is involved in the homeostasis between the human microbiome and colonising microorganisms. These include the fungal family *Malassezia* spp. [8,9]. These lipophilic fungi require an alkaline pH for growth. They usually colonise the skin from puberty onwards. The skin changes seen in AD allow *Malassezia* spp. to colonise the skin more easily. Changes in the immune system observed in AD skin, with decreased cathelicidins (LL37) and decreased β-defensins, also favour the colonisation of this fungus. Through mannan receptors expressed on dendritic cells, *Malassezia* spp. proteins are recognised by the innate immune system [10]. In addition, colonization of *Malassezia* spp. on the skin induces the release of cytokines, such as TNF-α, IL1β, and IL18 (without IL12). All this leads to antigen presentation to Th2 lymphocytes, with the release of IL13 [11]. This cytokine maintains homeostasis between the organism and colonisation by *Malassezia* spp. Blocking the Th2 pathway with drugs such as dupilumab induces an immune shift with increased activation of the Th17/Th22-mediated pathway. It has been shown in mouse models that overexpression of Th17/Th22 mediates the inflammatory hypersensitivity reaction to *M. furfur* fungal proteins [12]. It is therefore not surprising that psoriasiform rashes are observed in AD patients treated with dupilumab [13]. Similarly, seborrheic dermatitis-like rashes may also be seen in this same patient profile. In our study, no psoriasiform eruptions were observed in patients with AD in either the dupilumab-treated or the non-dupilumab-treated subgroups.

Although the pilot study was initially designed with two groups for comparison, the DS and the healthy group, we soon observed that the determination of IgE specific for *Malassezia furfur* in DS patients was negative in all patients. DS is considered to be an inflammatory dermatosis in response to this fungus, triggered by a mechanism different from that observed for IgE-mediated sensitization. No patient in the DS group showed IgE positivity specific for *Malassezia furfur*. The determination of this biomarker could be considered an interesting analytical tool to differentiate those cases of AD with H&N pattern from true DS. In facial DS, decreased diversity of the microbiome has been demonstrated with an increased *Malassezia* population [14]. This change in the skin microbiome is responsible for the inflammatory reaction mediated mainly by the innate immune system. The application of ketoconazole cream reduces the colonisation of *Malassezia furfur* and thus restores the microbiome.

*Malassezia* spp.-induced H&N pattern is characterised by seborrheic eczematous lesions in classic areas (ciliary, nasolabial fold, anterior cervical face), as well as on the scalp. It usually worsens with sweating and shows no improvement with conventional topical anti-inflammatory treatments (corticosteroids and topical calcineurin inhibitors). To date, the use of dupilumab has not been associated as a risk factor for this condition. However, in our study, we found a significant relationship between dupilumab, H&N AD pattern, and *Malassezia furfur*-specific IgE positivity.

Another interesting aspect to note was the location of the lesions in the group of patients with AD without sensitization to *Malassezia furfur* fungus. None of the patients had lesions on the head and/or neck. All showed a classic flexural pattern, with involvement of the popliteal and antecubital folds.

Regarding treatment, there are published cases in the literature with variable responses with fluconazole 150 mg/weekly [15]. In our experience, itraconazole 100 mg/12 h for 3 weeks showed a full or almost full response. The antifungal and anti-inflammatory effect of itraconazole may account for this successful therapeutic response [16]. In mild H&N AD cases, ketoconazole 2% cream every 12 h may be indicated, with satisfactory responses in these patients [17]. Oral itraconazole and topical ketoconazole may be combined when the clinical situation requires it—mainly patients with more extensive lesions. Overlapping cases with eczematous lesions may require combination with topical anti-inflammatory treatments, such as corticosteroids or topical calcineurin inhibitors.

Other possible etiologies should be taken into account in the workup of patients with H&N pattern AD. Palpebral involvement suggests allergic contact dermatitis, which requires patch testing. Facial erythematous patches may represent the specific picture induced by dupilumab (dupilumab-associated head and neck dermatitis (DAFND). Rosaceiform/demodicosis rashes, the pathophysiology of which is also mediated by Th2 pathway blockade and polarization towards Th17 [18], should also not be forgotten. The possibility of overlap between all these entities is possible, so it is necessary to be familiar with the clinical clues to properly approach the treatment of each patient. The main diseases that comprise the differential diagnosis of H&N AD pattern can be found in Table 3.

For the study of sensitization to *Malassezia* spp., there are other methods besides the determination of specific IgE. Studies with prick tests or patch tests are available [19]. However, in favour of serum specific IgE, we can point out that there are more published studies on this method and that it offers the advantage of being more accessible for the physician, without extraordinary time consumption for the patient. The main limitation of our study is the small number of patients included. The study was designed as a pilot study with patients registered during the recruitment period.

In conclusion, we present the study of sensitization to *M. furfur* in patients with AD and H&N pattern. The results are in line with our study objective, where sensitization to *Malassezia furfur* in patients with atopic dermatitis may be a cause of H&N pattern. Risk factors to consider include high total IgE levels, sensitization to pneumoallergens, CRP levels, and receiving dupilumab treatment. The most appropriate treatment seems to be the use of oral itraconazole and/or ketoconazole cream. We can add that the determination of *Malassezia furfur*-specific IgE can be considered a useful biomarker for diagnosis and therapeutic indication in H&N AD pattern.

## Figures and Tables

**Table 1:** Demographic, clinical, and serological characteristics.

	Atopic Dermatitis	Seborrheic Dermatitis	Healthy Controls
n	25	14	19
Age, years (mean ± SD)	31.92 ± 10.26	38.64 ± 17.08	36.58 ± 13.32
Sex ratio (M/F) *	0.48	0.79	0.63
Weight (mean ± SD)	73.24 ± 16.22	82.86 ± 4.88	72.11 ± 9.78
H&N involvement *	68%	100%	0%
Asthma history *	52%	0%	5%
Pneumoallergens history *	88%	15%	0%
Total IgE, IU/mL (mean ± SD) *	2210.96 ± 3260.30	241.37 ± 481.33	36.3 ± 54.56
*M. furfur* IgE > 0.35 (Ku.arb./L) *	80%	0%	0%
Total LDH level (U/L)	211.44 ± 53.62	205.33 ± 35.20	189.83 ± 21.71
Total CRP level (mg/L)	1.44 ± 0.82	3.08 ± 2.40	1.96 ± 0.99

* Statistically significant differences (*p* < 0.05).

**Table 2:** Subgroups of DA patients according to dupilumab treatment.

	Dupilumab	No Dupilumab
n	15	10
Age, years (mean ± SD)	34.33 ± 10.97	28.30 ± 8.33
Sex ratio (M/F)	0.60	0.30
Weight (mean ± SD) *	79.53 ± 16.42	63.80 ± 10.84
H&N involvement *	100%	20%
Asthma history	47%	60%
Pneumoallergens history	87%	90%
Total IgE, IU/mL (mean ± SD)	1772.48 ± 3053.84	2868.67 ± 3610.73
*M. furfur* IgE > 0.35 (Ku.arb./L)	93%	60%
Total LDH level (U/L)	201.93 ± 22.76	225.7 ± 80.53
Total CRP level (mg/L)	1.47 ± 0.92	1.40 ± 0.71

* Statistically significant differences (*p* < 0.05).

**Table 3:** Differential diagnosis of H&N atopic dermatitis pattern.

Dermatological Diseases	Clinical Features	Diagnostic Approach
Dupilumab facial redness	Fixed erythema located on the face or neck (may also occur extrafacially) Usually unilateral Resistant to corticosteroids and/or topical calcineurin inhibitors Patient on dupilumab treatment	Clinical Temporal correlation between dupilumab initiation and onset of erythema red facial erythema
Seborrhoeic dermatitis	Pityriasiform lesions (whitish “dry” scale) over orange erythema on nasolabial folds, ciliary area, beard or sideburn area, scalp or external auditory canal.	Clinical
Rosacea	Persistent malar erythema or with flushing exacerbations Telangiectasias of different calibre Papules and/or pustules	Clinical Dermoscopy Microscopic examination of *Demodex folliculorum* with tape test, scraping, etc.
Demodicosis	Facial itching and/or burning sensation (especially on the cheeks) Erythematous dilatation of facial follicular openings seen on dermoscopy	Clinical Dermoscopy Microscopic examination of Demodex folliculorum with tape test, scraping, etc.
Dermatitis perioralis	Monomorphous papular rash localised in the perioral region Asymptomatic	Clinical
Allergic contact dermatitis	Erythematous, scaly, very pruritic rash. Special patterns: palpebral, hairline, lateral facial and cervical sides, usually symmetrical	Patch testing with standard and specific series
Airborne dermatitis	Facial and cervical erythematous-squamous rash, with involvement of the eyelids, retroauricular, and submandibular areas. Respect of the nasal triangle	Allergological study by means of: - Specific IgE in serum for pneumoallergens. - Allergen specific prick test
*Malassezia* head/neck dermatitis	Facial and cervical rash mainly localised in seborrheic-like areas Patients on dupilumab treatment Age from adolescence onwards (infrequent in childhood) Refractoriness to topical corticosteroids and/or topical calcineurin inhibitors May appear de novo or be persistent after starting dupilumab treatment	*Malassezia furfur*-specific IgE serum (>0.35 IU/mL) Clinical response to treatment with topical and/or oral antifungals
Topical steroid withdrawal	More frequent in women Prolonged use of topical corticosteroids Erythematous hypersensitive skin appearance Local sensation of itching, heat, pain, burning, etc.	Anamnesis (confirmation of chronic use of topical corticosteroids on the face) Clinical

## Data Availability

Not applicable.
